# Supplementary figures and images for: Phase separation-mediated actin bundling by the postsynaptic density condensates
Source: eLife. 2023 Jun 15;12:e84446. doi: 10.7554/eLife.84446 (PMC10322149; doi:10.7554/eLife.84446)

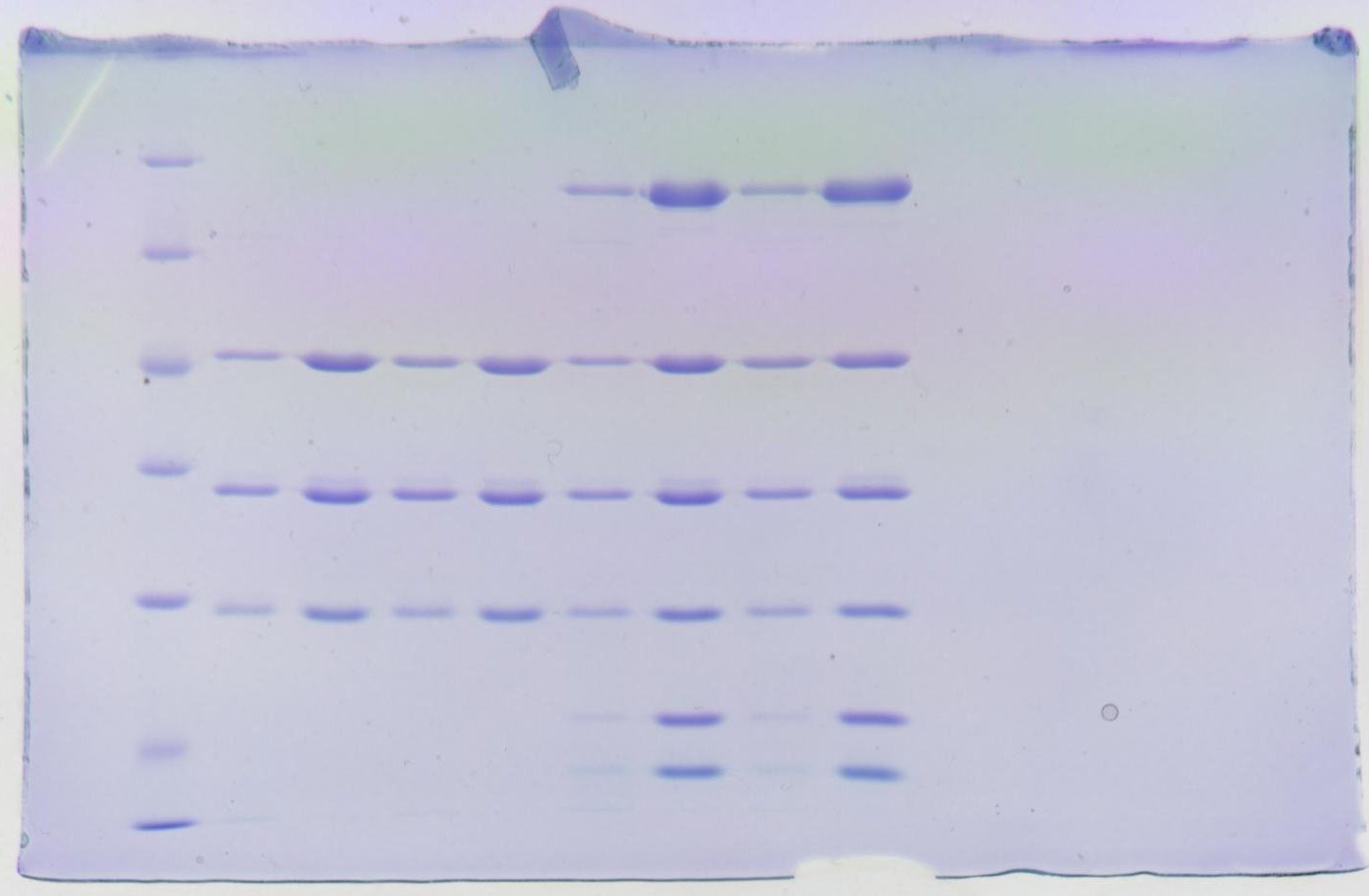

Supplement: Figure 3—source data 1. [file elife-84446-fig3-data1.jpg]

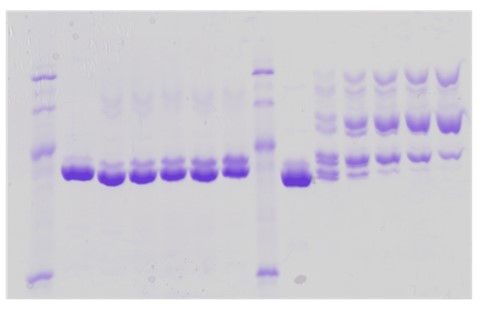

Supplement: Figure 4—source data 1. [file elife-84446-fig4-data1.jpg]

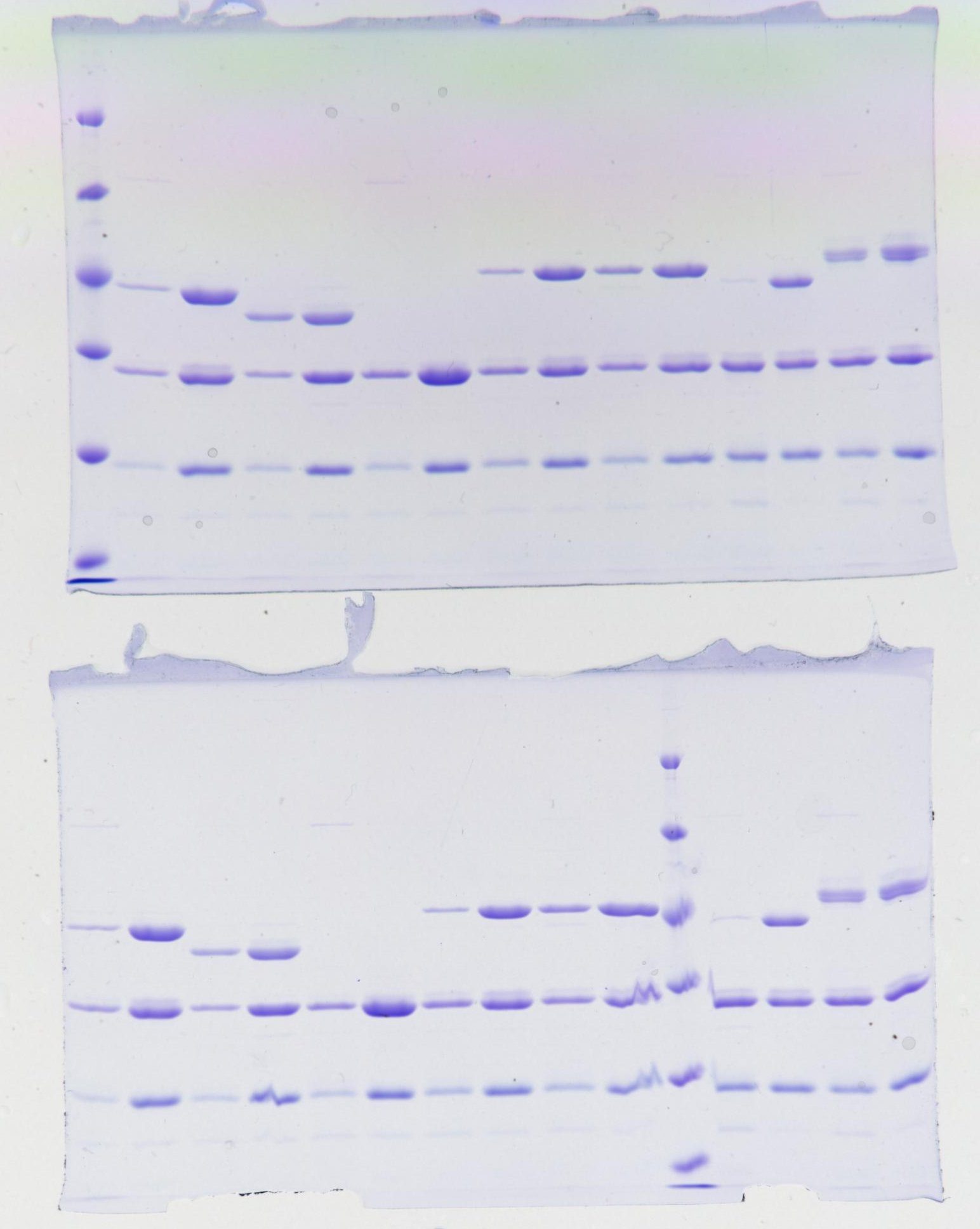

Supplement: Figure 4—source data 2. [file elife-84446-fig4-data2.jpg]

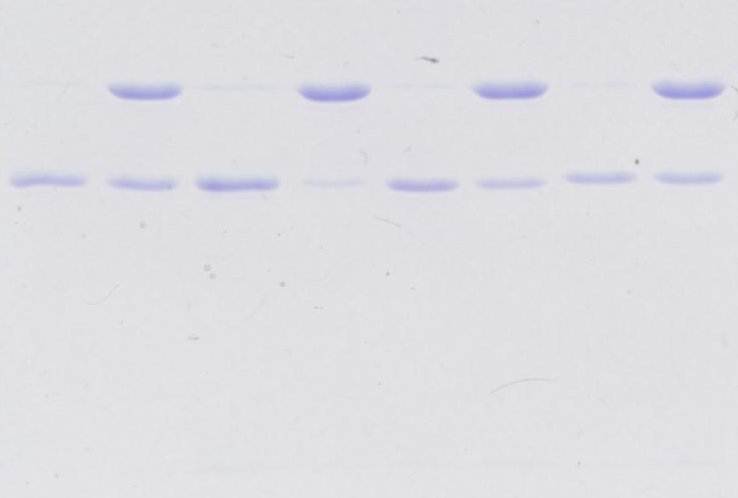

Supplement: Figure 5—source data 1. [file elife-84446-fig5-data1.jpg]

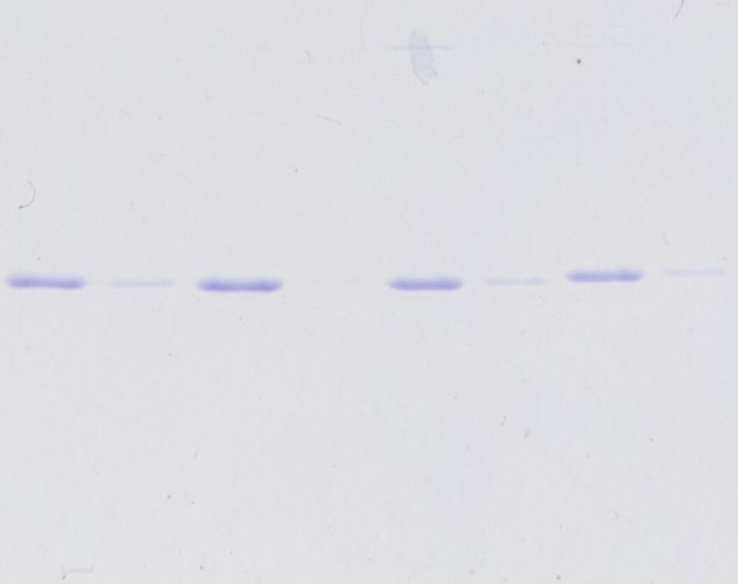

Supplement: Figure 5—source data 2. [file elife-84446-fig5-data2.jpg]
